# Towards Smart Homes Using Low Level Sensory Data

**DOI:** 10.3390/s111211581

**Published:** 2011-12-12

**Authors:** Asad Masood Khattak, Phan Tran Ho Truc, Le Xuan Hung, La The Vinh, Viet-Hung Dang, Donghai Guan, Zeeshan Pervez, Manhyung Han, Sungyoung Lee, Young-Koo Lee

**Affiliations:** OS Lab, Department of Computer Engineering, Kyung Hee University, Yongin-Si, 446-701, Korea; E-Mails: asad.masood@oslab.khu.ac.kr (A.M.K.); pthtruc@hotmail.com (P.T.H.T.); xuanhung_le@urmc.rochester.edu (L.X.H.); vinhlt@oslab.khu.ac.kr (L.T.V.); dangviethung@oslab.khu.ac.kr (V.-H.D.); donghai@oslab.khu.ac.kr (D.G.); zeeshan@oslab.khu.ac.kr (Z.P.); smiley@oslab.khu.ac.kr (M.H.); yklee@khu.ac.kr (Y.-K.L.)

**Keywords:** accelerometer, location sensor, video sensor, u-healthcare, activity recognition

## Abstract

Ubiquitous Life Care (u-Life care) is receiving attention because it provides high quality and low cost care services. To provide spontaneous and robust healthcare services, knowledge of a patient’s real-time daily life activities is required. Context information with real-time daily life activities can help to provide better services and to improve healthcare delivery. The performance and accuracy of existing life care systems is not reliable, even with a limited number of services. This paper presents a Human Activity Recognition Engine (HARE) that monitors human health as well as activities using heterogeneous sensor technology and processes these activities intelligently on a Cloud platform for providing improved care at low cost. We focus on activity recognition using video-based, wearable sensor-based, and location-based activity recognition engines and then use intelligent processing to analyze the context of the activities performed. The experimental results of all the components showed good accuracy against existing techniques. The system is deployed on Cloud for Alzheimer’s disease patients (as a case study) with four activity recognition engines to identify low level activity from the raw data captured by sensors. These are then manipulated using ontology to infer higher level activities and make decisions about a patient’s activity using patient profile information and customized rules.

## 1. Introduction

As living standards increase, people are more interested in their health and desire to have a healthy life. As a result, healthcare system costs are increasing worldwide. According to the Organization of Economic Cooperation and Development (OECD; http://www.oecd.org/statsportal/0,3352,en_2825_293564_1_1_1_1_1,00.html) health data, total health spending accounted for 15.3% of GDP in the United States in 2006, the highest share in the OECD. The corresponding number in Korea is 6.4%. To maintain the quality and availability level of life care services with minimal cost, a powerful, flexible, and cost-effective infrastructure for life care services is needed, such as ubiquitous life care (u-Life care).

Because of its elasticity, scalability and pay-as-you-go model [1], Cloud Computing can potentially provide huge cost savings, flexibility, high-throughput, and ease of use for life care services. To take advantage of these characteristics of Cloud Computing, we have developed a platform architecture, called Secured Wireless Sensor Network (WSN)—integrated Cloud Computing for u-Life Care (SC^3^) [2,3]. Different wireless sensors are deployed that collect real-time data and transmit it to the Cloud Server through a Cloud Gateway. Based on the data collected by different sensors, SC^3^ provides real-time healthcare and safety monitoring services, information sharing and exchange capability, emergency connection services, and patient monitoring and care services.

Existing AR systems are based on simple conditions and actions [4], do not use context information or in some cases use imperfect context information [5] where the result of a system is unpredictable. Research on reminders for elders to perform daily life activities [6] is getting more focus. These are plan-based approaches to decide when and how to prompt subjects effectively. The focus is on time-based activities. To overcome the limitations of this system, a Location-based reminder system was introduced [7], where the key element for reminders is the location of the subject. In fact, context for reminders is more important than simple location or time and context includes both location and time as subsets. HYCARE [8] is a reminder system that takes context into consideration and develops a novel scheduling mechanism that can coordinate various reminder services and remedy possible conflicts. The system discussed in [4] is a more realistic system that uses ontology to incorporate context in intelligent processing of the collected information. They also focus on information collected from sensors like smoke detectors, GPRS modems, infrared controls and X10 appliances that actually facilitate more in-home care for the person than out-of-home healthcare. It is based on the Event-Condition-Action (ECA) model.

The above discussed systems do not use real-time activities or only use one type of real-time activity to generate reminders or make decisions. Context is limited to time and location, which results in inflexible system behavior. These systems can mostly be categorized as reminder systems or homecare systems. Existing systems do not facilitate healthcare, which is the most important aspect. Real-time healthcare service provisioning raises very important questions: (1) How to more accurately recognize user activity and situations? (2) How to avoid the possibility of missing context information? (3) How to provide real-time personalized services? (4) How to support increasing needs for storage and computation for data and service provisioning?

To answer these questions and provide reliable real-time healthcare services, we have developed SC^3^. One of the main components of SC^3^ is the Human Activity Recognition Engine (HARE), presented in [3] with some initial results. This component is necessary and important because in order to provide improved daily medical care and real-time reaction to medical emergencies, identifying the patient’s activities, so-called Activity Recognition (AR) and context-aware manipulation is a prerequisite. However, AR is a collaborative activity; even the use of only video-based AR can be complex due to abrupt object motion, noisy images, the non-rigid or articulated nature of the human body, partial and full object occlusions, scene illumination changes, and real-time processing requirements. The process is further complicated because a person will not perform an activity in the same way each time; the user may often perform multiple activities simultaneously; every individual will most probably perform activities differently; and every building will have a different layout and sets of deployed sensors. A person’s activities will change over time as his medical condition changes. To handle all these situations and activities, we use multiple activity recognition techniques and model these activities and situations in order to have a reliable mechanism for activity recognition and situation analysis.

Humans rely on several modalities including the five classical senses and other senses such as thermoception (temperature) and equilibrioception (balance and acceleration) together with context information such as location and time for everyday tasks. Currently, to the best of our knowledge, there is no systematic way to integrate multiple modalities such as vision with motion, environment, location, and time to infer human intentions. Our focus in this paper is on the HARE component of the SC^3^ architecture we developed [2]. The proposed HARE can enhance capabilities and provide tremendous value for smarter service provisioning and recommendation. Considering context during decision making is an important factor [5]. For implementation of HARE, we use all possible sources of information to avoid any possibility of missing information or avoid using imperfect context information. Sensors are deployed to collect real-time data about the person’s activities and the environment information. Then with the help of ontology (containing expert knowledge in the medical domain) these detected activities are intelligently manipulated to infer higher level activities and complete the situation analysis. The experimental results for four components of our HARE architecture, namely video-based activity recognition, sensor-based activity recognition, location tracking, and context-aware activity manipulation have also verified our claims. The results of proposed procedures for activity recognition and manipulation are very encouraging in terms of accuracy. At the end, we provide a case study of our system demonstration for Alzheimer’s disease patients. The demonstration is successful for real-time activities that Alzheimer’s patients perform or should perform.

This paper is arranged as follows: Section 2 includes a detailed description of the proposed HARE architecture and the methodology that is followed to achieve the objectives. In Section 3, detailed results for four components of HARE are presented. The discussion section (Section 4) includes a discussion of the results, the system application for Alzheimer’s disease patients. Finally we discuss future directions and applications in Section 5.

## 2. Proposed HARE Architecture

The Human Activity Recognition Engine (HARE) is designed to provide an accurate and robust human Activity Recognition (AR) and Manipulation system to better understand living situations and decision making for u-healthcare environments. Many research groups are addressing the problem of determining the Activities of Daily Life (ADL), where they mostly focus on one or a few activities, using one or a few techniques, and often lack robustness to determine those activities in complex situations. Our proposed AR system incorporates video, accelerometer, location, and physiological data to improve the robustness and scope of ADL capabilities. For example, it will categorize many complex activities such as taking pills, doing exercises, watching TV or undergoing serious situations like eating, tooth brushing, falling down, and heart attacks. To provide intelligent service recommendations and situation analysis, context information (expert knowledge) is used to reduce the chance of using incorrect information from heterogeneous devices operating in that environment.

**Figure 1.**
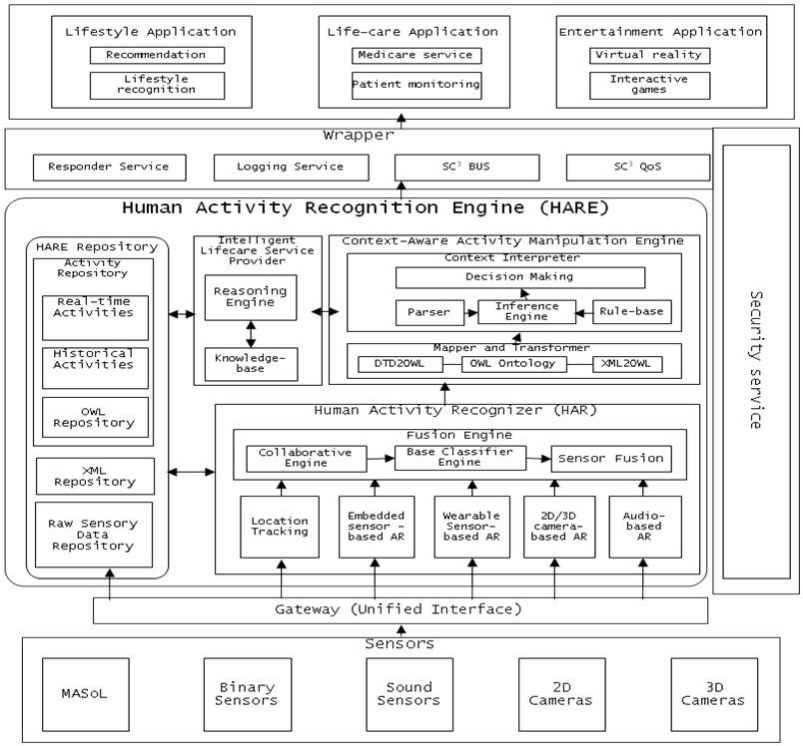
Architecture of Human Activity Recognition Engine (HARE).

The core of SC^3^ is a Human Activity Recognition Engine (HARE) as shown in Figure 1. HARE is composed of various sub-components such as:

- Location Tracking: tracking a person’s location in a given environment. This module is developed for both indoor activities and outdoor activities.
- Activity Recognizer: This component includes embedded, wearable, 2D camera- and 3D camera-based activity recognition engines to recognize human activities. These sensors are the outer world agents of HARE. They collect human activity information and use the training set to recognize human activity.
- Fusion Engine: Human activities are recognized using different input modalities (sensors), so at certain times, two different modalities may produce different activity labels for a given activity. To handle this situation and reach a consensus, Fusion Engine is used to aggregate the activity and classify it. It is important to collaborate between different activity recognition engine approaches. It is necessary to increase the accuracy of activity recognition. For example, if embedded sensor-based AR detects a person is taking medicine with 70% accuracy, and 2D video-based AR detects the person is taking medicine with 80% accuracy, then the collaborator can ensure that the person is taking medicine. Standardization is required for the collaboration accuracy mechanism.
- Schema Mapping and XML Transformer: Once an activity is recognized by the activity recognition components, it is represented in XML representation format for communication with other modules and for manipulation. To transform activity output from XML into a machine-understandable and flexible OWL format, the Schema Mapping and XML Transformer component is developed.
- Context-Aware Activity Manipulation Engine (CAME): This component infers high level activities or makes decisions in accordance with each human activity (low level activity) based on human (patient) profile information and situation analysis.
- Intelligent Lifestyle Service Provider (*i*-LiSP): This component recommends the most suitable services to the user based on the activities that the user (patient) has performed over time.
- Wrapper: This component is used to secure the data and services of HARE deployed on the Cloud. It handles all requests for services from third party applications or users from the outer world and routes the complete request and the response to the request.
- HARE Repository: This is the backbone of HARE; it stores raw data collected by sensors and cameras, stores real-time activities recognized by activity recognition engines, activity history and activities in machine understandable format (e.g., OWL) to infer high level activities.
- Mobile Activity Sensor Logger (MASoL): Though it is not the focus of this article, it is important to mention that we have successfully developed a Mobile Activity Sensor Logger (MASoL) which serves in the infrastructure layer under HARE to collect and monitor human and environment information. Sensors such as accelerometers, geomagnetic, gyroscopes and many others are required to collect diverse information. To collect those data from the human body, one should have sensors attached to the body all day to accumulate data. MASoL is our own sensor logger, which contains 13 axis sensors to gather raw activity data and store it. To prevent inconvenience, all kinds of sensors are integrated into one board with flexible software architecture to improve data efficiency.

For the Human Activity Recognizer (HAR), we have been researching different AR approaches such as embedded sensor-based AR, wearable sensor-based AR, 2D camera-based AR, and 3D camera-based AR. Each approach has pros and cons in different situations. In the wearable sensor-based approach, a single sensor is attached to each person. It supports gyroscope and 3-axis accelerometer measurement. An activity is predicted or inferred based on the gyroscope and accelerometer information. Activity history is also used to increase the accuracy of current activity detection. We have proposed a novel structure of semi-Markov Conditional Random Fields and a fast algorithm for training, making the model able to take advantage of inter-relationships and the duration of activities to improve the accuracy and reduce the time needed to classify performance [9]. Even though low accuracy may result in some particular cases, we have shown that our approach obtains better results than others.

In the embedded sensor-based approach, a group of sensors are attached to objects and the surrounding environment, for example on a wall, on monitor, on an electronic notepad, on a medicine tray or on a patient bed. Normally, when a person performs an activity, the person must use a number of objects/tools. Whenever an object/tool is used, the attached sensor will report a signal to the central server. Using a sequence of these signals, we can predict or infer what the user is doing. We have shown that it is possible to train an activity classifier using such knowledge. One of the major advantages of such a technique is that it eliminates the amount of human effort required for labeling activities while still achieving high recognition accuracy with good performance. Another advantage of this technique is that it is possible to label thousands of activities within a very short period of time [9]. We have also presented a multi-level Naive Bayes (NB) based activity classifier with a smoothing technique to improve the accuracy of activity classification.

Video-based approaches are new methods of recovering human body posture from the stereo information acquired by the stereo camera [10]. The technique is formulated in a unique probabilistic framework and implemented via two stage optimization of the Expectation-Maximization (EM) algorithm. With the combination of various potentials in our probabilistic model, including the smoothness term, the image likelihood, the reconstruction error between the model and the observation, and the geodesic distance, our method can determine the appearance of the body parts. The locations of the body parts are integrated in the co-registration process to estimate human body postures. However, the occlusions of one body part by the others may affect body part detection.

Semantic Data Transformation transforms activity output into domain knowledge so that the computer can easily understand and process it efficiently. It allows the automatic mapping of XML to OWL Full ontology. Our procedure has several advantages over existing methods. First, while transforming all the elements of an XML document into OWL, our algorithm retains the original structure and captures the implicit semantics in the structure of the XML document. Second, elements in XML are classified in classes or properties based on their definition and detailed descriptions in DTD. This makes the result independent of users’ opinions. DTD is used for defining the XML structure, XML for describing data, OWL for providing definition and relationship among the data items. These relationships are later used to infer high level activities from low level activities.

Our HARE is designed in such a flexible manner that its client can easily communicate with it from small hand held devices such as sensors, PDA’s, or cell phones. Various entertainment applications such as online games and console games can use HARE. HARE is deployed on a computing cloud to reduce data costs. The cloud server (SC^3^) provides more storage and computational capability than individual computer systems and servers. Software and operating systems updates need not be managed and the information gathered is available anywhere.

## 3. Implementation and Results

One of the main purposes of u-Life care is to enable people to live longer independently by early detection and prevention of chronic diseases and disabilities. Computer vision, wireless sensor networks (WSN), and body networks are emerging technologies that promise to significantly enhance medical care for seniors living at home or in assisted living facilities. With these technologies, we can collect video, physiological, and environmental data, identify individuals’ activities of daily living (ADL), and improve daily medical care as well as real-time responses to medical emergencies.

To achieve this, accurately identifying individuals’ ADL via activity recognition (AR) is of vital importance. It is also a significant challenge. For instance, video-based AR is complex [10]. In this article, we discuss the results of video-based activity recognition, embedded sensor-based activity recognition, and location tracking of subjects. The recognized activities are then forwarded to a context-aware activity manipulation engine (CAME) to infer higher level activities.

### 3.1. Video Based Activity Recognition

The accuracy of the video-based AR depends significantly on the accuracy of human body segmentation. In the field of image segmentation, first introduced by Kass *et al*. [11], the active contour (AC) model has attracted much attention. Recently, Chan and Vese (CV) proposed a novel form of AC based on the Mumford and Shah functional for segmentation and the level set of the framework. [12] The CV AC model utilizes the difference between the regions inside and outside of the curve, making itself one of the most robust and thus widely used techniques for image segmentation. Its energy functional is:

(1) $$F\left(C\right)=\int_{\mathrm{in}\left(C\right)}{\left|I\left(x\right)-c_{\mathrm{in}}\right|}^{2}dx+\int_{\mathrm{out}\left(C\right)}{\left|I\left(x\right)-c_{\mathrm{out}}\right|}^{2}dx$$

where **x** ∈ Ω (the image plane) ⊂ *R*^2^, *I*: Ω → Z is a certain image feature such as intensity, color, or texture. *c_in_* and *c_out_* are respectively the mean values of the image feature inside [*in*(*C*)] and outside [*out*(*C*)] the curve *C*, which represent the boundary between two separate segments. By considering image segmentation as a clustering problem, we observe that this model forms two segments (clusters) such that the differences within every segment are minimized. However, the global minimum of the above energy functional does not always guarantee desirable results, especially when a segment is highly inhomogeneous, as a human body is, since the purpose of the calculation is to minimize the dissimilarity within each segment without considering the distance between different segments. Our methodology incorporates an evolving term based on the Bhattacharyya distance [13] to the CV energy functional [14] such that not only the differences within each region are minimized but the distance between the two regions is maximized as well. The proposed energy functional is:

(2) $$E_{0}\left(C\right)=\beta F\left(C\right)+\left(1-\beta \right)B\left(C\right)$$

where *β* ∈ [0,1] and $B\left(C\right)\equiv B=\int_{𝒵}\sqrt{p_{\mathrm{in}}\left(z\right)p_{\mathrm{out}}\left(z\right)}\mathrm{dz}$ is the Bhattacharyya coefficient with:

(3) $$p_{\mathrm{in}}\left(z\right)=\frac{\int_{\Omega }\delta \left(z-I\left(x\right)\right)H\left(-\varphi \left(x\right)\right)dx}{\int_{\Omega }H\left(-\varphi \left(x\right)\right)dx}$$

(4) $$p_{\mathrm{out}}\left(z\right)=\frac{\int_{\Omega }\delta \left(z-I\left(x\right)\right)H\left(\varphi \left(x\right)\right)dx}{\int_{\Omega }H\left(\varphi \left(x\right)\right)dx}$$

where ϕ: Ω→*R* is the level set function, and *H*(·) and *δ*(·) ≜ *H*′(·) are the Heaviside and the Dirac functions, respectively. Note that the Bhattacharyya distance is defined by [−log *B*(*C*)] and the maximization of this distance is equivalent to the minimization of *B*(*C*). Also note that to be comparable to the *F*(*C*) term, in our implementation, *B*(*C*) is multiplied by the area of the image because its value is always within the interval [0,1] whereas *F*(*C*) is calculated based on the integral over the image plane. In general, we can regularize the solution by constraining the length of the curve and the area of the region inside it. Therefore, the energy functional is defined by:

(5) $$E\left(C\right)=\gamma \int_{\Omega }\left|\nabla H\left(\varphi \left(x\right)\right)\right|dx+\eta \int_{\Omega }H\left(-\varphi \left(x\right)\right)dx+\beta F\left(C\right)+\left(1-\beta \right)B\left(C\right)$$

where *γ* ≥ 0 and *η* ≥ 0 are constants.

The intuition behind the proposed energy function is that we seek a curve which (1) is regular (the first two terms) and (2) partitions the image into two regions such that the differences within each region are minimized (*i.e*., the *F*(*C*) term) and the distance between the two regions is maximized (*i.e*., the *B*(*C*) term).

The level set implementation for the overall energy functional can be derived as:

(6) $$\frac{\partial \varphi }{\partial t}=\left|\nabla \varphi \right|\left\{\begin{array}{c} \gamma \kappa +V_{0}+\beta \left[{\left(I-c_{\mathrm{in}}\right)}^{2}-{\left(I-c_{\mathrm{out}}\right)}^{2}\right]\\ -\left(1-\beta \right)\left[\frac{B}{2}\left(\frac{1}{A_{\mathrm{in}}}-\frac{1}{A_{\mathrm{out}}}\right)+\frac{1}{2}\int_{𝒵}\delta_{0}\left(z-I\right)\left(\frac{1}{A_{\mathrm{out}}}\sqrt{\frac{p_{\mathrm{in}}}{p_{\mathrm{out}}}}-\frac{1}{A_{\mathrm{in}}}\sqrt{\frac{p_{\mathrm{out}}}{p_{\mathrm{in}}}}\right)\mathrm{dz}\right] \end{array}\right\}.$$

where *A_in_* and *A_out_* are respectively the areas inside and outside the curve *C*.

**Figure 2.**
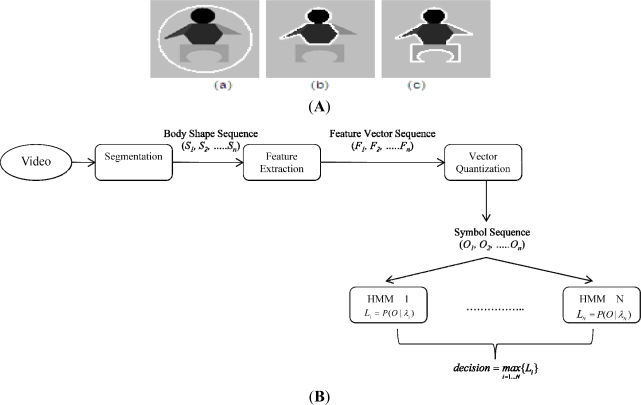
(**A**) Shows sample segmentation of inhomogeneous body-shape object using active contours. (a) Initial contour; (b) result of CV AC [12]; and (c) result of our approach. (**B**) Shows the architecture of our approach for motion feature extraction and recognition.

As a result, the proposed model can overcome the CV AC’s limitation regarding segmenting inhomogeneous objects as shown in Figure 2(c), resulting in a body detector more robust to illumination changes. After obtaining a set of body silhouettes segmented from a sequence of images, we apply ICA (independent component analysis) [15,16] to get the motion features of that sequence. ICA focuses on the local feature rather than on global features as in PCA (principal component analysis) ([16], chapter 6). The extracted features are then symbolized using vector quantization algorithms such as K-mean clustering [17]. Finally, a symbol sequence is used to generate a codebook of vectors for training and recognition.

The overall architecture of proposed framework is shown in Figure 2(B), where *T* represents the number of testing shape images, *N* number of trained HMMs, and *L* likelihoods. For evaluating video-based activity recognition, a publicly available dataset [10] containing video clips of nine activities, namely “bend”, “jack” (jumping-jack), “jump” (jumping forward on two legs), “run”, “side” (gallop sideways), “skip”, “walk”, “wave1” (wave-one-hand), and “wave2” (wave-two-hands) were used. Each activity was performed by nine different people. Each clip was down-sampled to produce two clips. By this method, we have, for each activity, nine clips for training and nine clips for testing. The lengths of the clips do not have to be identical, which is a major advantage of the HMM approach. The video frames were resized to 100 × 70. The recognition rates of our system are summarized in Table 1.

### 3.2. Sensor Based Activity Recognition

Based on existing work [18], we develop our own recognition technique, which is called “semi-Markov Conditional Random Fields (semiCRF)” [9]. Furthermore, we propose a novel algorithm which helps to reduce the complexity of the training phase a factor of more than 10 compared with original work. In our model, we assume that:

(7a) $$X=\left\{x_{1},x_{2},\ldots x_{T}\right\}$$

and:

(7b) $$Y=\left\{y_{1},y_{2},\ldots y_{T}\right\}$$

are the input signals and input label respectively. Our goal is to optimize the model parameter so that P(Y|X) is maximized. With conventional conditional random fields P(Y|X) is calculated by:

(8) $$P\left(Y|X\right)=\frac{\prod_{t=1}^{T}\Psi \left(y_{t-1},y_{t},X\right)}{Z_{X}}$$

(9) $$\Psi \left(y_{t-1},y_{t},X\right)=e^{W^{T}F\left(y_{t-1},y_{t},X\right)}$$

(10) $$Z_{X}=\sum_{Y^{\prime }}\prod_{t=1}^{T}\Psi \left(y_{t-1}^{\prime },y_{t}^{\prime },X\right)$$

where F is a column vector of feature functions (which are often delta functions), W is a column vector of model parameters, and ψ are so-called potential functions. Z_X_ (normalization factor) is computed by using a forward or backward algorithm. However, conventional CRF is limited to the Markov assumption, which is not able to model the duration of activity as well as the interdependency among activities. To overcome these, the semi-Markov model was first introduced in [18], by defining a new state as s_i_ = (y_i_, b_i_, e_i_) where s_i_ is the i^th^ state and y_i_, b_i_, and e_i_ in that order are label, begin and end time of the state. For example, given an input label sequence Y = (1, 1, 2, 2, 2, 3, 4, 4), the semi-Markov state sequence is s_1_ = (1, 1, 2), s_2_ = (2, 3, 5), s_3_ = (3, 6, 6), s_4_ = (4, 7, 8). However, the proposed semi-Markov Conditional Random Fields (semiCRF) are unsuitable for activity recognition because of the presence of the so-called null activities, which are the activities that we do not intend to recognize. For example, if we have a sequence of activities Y = (eating, eating, cleaning, cleaning, having tea, having tea), and we just want to detect eating and drinking activities, cleaning is a null activity. In such a case, the method in [18] will construct a semi-Markov sequence S = {(eating, 1, 2), (cleaning, 3, 4), (having tea, 5, 6)}. In this sequence, the relationship between eating and having tea cannot be modeled because of the Markov assumption. To resolve the problem, our main idea is to eliminate the null activities from the sequence, and hence our semi-Markov sequence is {(eating, 1, 2), (having tea, 5, 6)}. Therefore, unlike the semi Markov model proposed in [18] to capture the long-range transitions among activities, a more general constraint b_i+1_ > e_i_ is used instead of b_i+1_ =e_i_ + 1. With these definitions, the potential function is rewritten as:

(11) $$\Psi \left(s_{i-1},s_{i},X\right)=\left(\begin{array}{c} e^{Q^{\mathrm{Tr}}\left(s_{i-1},s_{i},X\right)}\times \\ e^{Q^{D}\left(s_{i-1},s_{i},X\right)}\times \\ e^{Q^{O}\left(s_{i-1},s_{i},X\right)} \end{array}\right)$$

where:

(12) $$Q^{\mathrm{Tr}}\left(s_{i-1},s_{i},X\right)=\sum_{y^{\prime },y}w^{\mathrm{Tr}}\left(y^{\prime },y\right)\delta \left(s_{i-1}.y=y^{\prime },s_{i}.y=y\right)$$

is a weighted transition potential function, w^Tr^(y′, y) is the weight of transition from y′ to y, and the delta function is defined as:

$$\delta \left(A\right)=\{\begin{array}{c} 1\;\text{if A is true}\\ 0\;\text{if A is false} \end{array}$$

Next, we define the weighted duration potential function as:

(13) $$\begin{array}{l} Q^{D}\left(s_{i-1},s_{i},X\right)=\sum_{y,d}G^{D}\left(y,d\right)\delta \left(s_{i}.y=y,d=s_{i}.e-s_{i}.b+1\right)\\ =\sum_{y,d}w^{D}\left(y\right)\frac{{\left(d-m_{y}\right)}^{2}}{{2\sigma }_{y}^{2}}\delta \left(s_{i}.y=y,d=s_{i}.e-s_{i}.b+1\right) \end{array}$$

where w^D^(y) is the weight of duration of the label y. m_y_ and σ_y_^2^ are the mean and variance of label y’s duration respectively, which are extracted from training data:

(14) $$Q^{O}\left(s_{i-1},s_{i},X\right)=\sum_{y,t_{1},t_{2}}\left(\begin{array}{c} G_{y}\left(y,t_{1},t_{2}\right)\times \\ \delta \left(s_{i}.y=y,s_{i}.b=t_{1},s_{i}.e=t_{2}\right)+\\ G_{\mathrm{IA}}\left(\mathrm{IA},t_{1},t_{2}\right)\times \\ \delta \left(s_{i-1}.e+1=t_{1},s_{i}.b-1=t_{2}\right) \end{array}\right)$$

is a weighted observation potential function, with:

(15) $$G_{y}\left(y,t_{1},t_{2}\right)=\sum_{t=t_{1}}^{t_{2}}\sum_{o}w^{o}\left(y,o\right)\delta \left(x_{t}=o\right)$$

and:

(16) $$G_{\mathrm{IA}}\left(IA,t_{1},t_{2}\right)=\sum_{t=t_{1}}^{t_{2}}\sum_{o}w^{o}\left(\mathrm{IA},o\right)\delta \left(x_{t}=o\right)$$

where w^o^(*y*,*o*) and *w^o^*(*IA*,*o*) in that order are the weights of the observation given that input symbol o is observed in a state with label *y* (label of expected activity, which is the activity that we want to recognize) and *IA* (label of unexpected activity, which is the activity that we do not intend to detect).

With the potential functions defined above, the training algorithm will search for parameter values which maximize the likelihood of the true labels, computed by:

(17) $$L\left(S|X\right)=\sum_{i=1}^{P}\left(\begin{array}{c} Q^{\mathrm{Tr}}\left(s_{i-1},s_{i},X\right)+\\ Q^{D}\left(s_{i-1},s_{i},X\right)+\\ Q^{O}\left(s_{i-1},s_{i},X\right) \end{array}\right)-\log\left(Z_{X}\right)$$

where:

(18) $$Z_{X}=\sum_{S^{\prime }}\prod_{i=1}^{P^{\prime }}\Psi \left(s_{i-1}^{\prime },s_{i}^{\prime },X\right)$$

The inference algorithm will find the most probable sequence (S) given the observation X as follows:

(19) $$S=\underset{S^{\prime }}{\text{argmax}}\;L\left(S^{\prime }|X\right)$$

**Figure 3.**
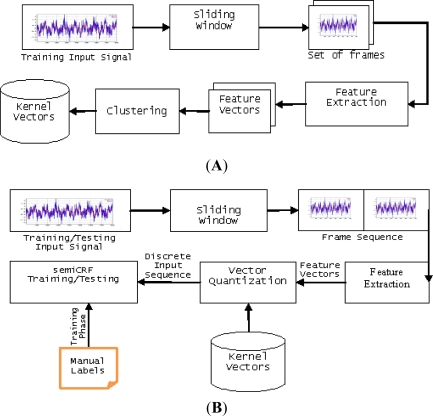
(**A**) Quantization module, where (**B**) Training and Testing module.

Making use of semi-Markov conditional random fields, we present here a block diagram of our recognition system as in Figure 3(A,B).

We proposed our algorithm for computing the gradients of the target function by extending [18]. It reduces the complexity of computing each gradient from O(NTM^2^D) to O(3TM(M+D)+NTD), where T, M, D, and N are the length of the input sequence, number of label values, and maximum duration of a label, and the number of gradients, respectively.

**Figure 4.**
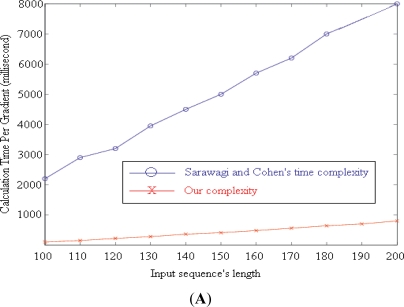

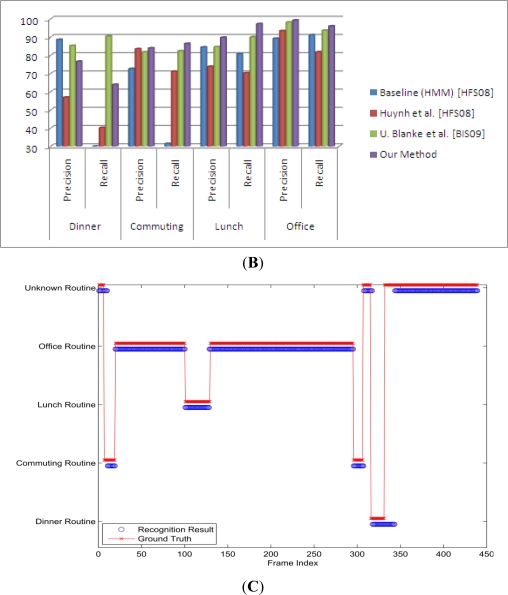
(**A**) Average time needed for computing 100 gradients against [18]; (**B**) Comparison of recognition results; (**C**) Single day recognized routines.

Figure 4(A) illustrates the calculation time required by the two algorithms to compute N = 100 gradients with M = 7, D = 256. T increases from 100 to 200. The blue line (upper line) shows the time needed by the original algorithm proposed by Sarawagi and Cohen [18]. Our time requirement is represented by the red line (lower line).

In our experiments, we used the dataset of long-term activities available online (http://www.mis.informatik.tu-darmstadt.de/data). Then we show our result and compare it to the original one. The dataset contains seven days of continuous data (except for sleeping time), measured by two triaxial accelerometers, one at the wrist and the other in the right pocket. The sensor’s sampling frequency was set at 100 Hz. The author calculated the mean value of every 0.4 s window; therefore the actual sampling frequency was about 2.5 Hz. In total, it has 34 labeled activities. A subset of 24 activities occurred during the routines and are grouped into five daily routines with their number of occurrences defined as dinner = 7, commuting = 14, lunch = 7, office work = 14, and unknown >50 [19].

In the experiments, we used a 50%-overlapped-sliding window, which has a length of 512 samples (about 3.41 min). To choose the right values for parameters, we conducted several experiments with different parameter values [9] then we chose the set of values, which produced the best results. Within the window, mean, standard deviation, and mean crossing rate are extracted from each signal. After that these values are combined with the time of frame to form a feature vector. Finally, we follow the leave-one-out cross validation rule to measure the result of recognition. The result can be seen in Figure 4(B,C) which demonstrates the recognition results together with the ground truth.

### 3.3. Location Tracking

The objective of location tracking or localization is to provide location information about the object of interest. Although video-based AR could perform location tracking, it fails in situations such as crowded tracked objects or because of privacy requirements (e.g., no cameras are allowed in bedrooms and bathrooms). Some approaches have been proposed using radio frequency (RF) [20,21] where the learning phase is taken and the result of inference is the location. In this work, we use a Neural Network instead of directly inferring the location, to infer the distances to the beacons and then use Push Pull Estimation (PPE) [22] to get the location. This technique is in fact developed for an outdoor environment, with high density of tracked objects and few beacons, the localization accuracy can be improved significantly. For the indoor tracking problem, it is still a good candidate solution because it is a successive refining method; because the result of the last inference is used as an initial guess for the next inference, which is not allowed in previous work.

**Figure 5.**
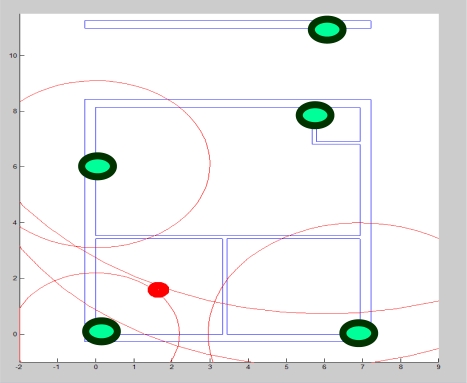
Test Bed for u-Life Care and deployment of Zio Access Point (WLB5254AP) to capture the location of subject (red dot).

For location tracking [20], we need to an environment with a pre-defined map of all the items in that environment. For this reason, we created the setting of our test bed center as shown in Figure 5. The test bed consists of three rooms and one corridor. The area is 11 m × 7 m. Beacons are the Zio Access Point (WLB5254AP) (shown in green in Figure 5) deployed to capture the signals of the subject location. The Radio Signal Strength (RSS) values from the Access Points (AP) are scanned by a mobile PDA (HP iPAQ) utilizing the WiFi Scanning driver of the PDA. Then these values are sent to the base station via BlueTooth and the base station forwards the data to the Cloud.

In the learning phase, the whole area is divided into a grid of 1 meter. We measured RSS from 5 APs (RSS vector):

(20) $$\mathrm{RSS}={\left[{\mathrm{RSS}}_{1}\;{\mathrm{RSS}}_{2}\ldots {\mathrm{RSS}}_{5}\right]}^{T}$$

where *RSS_i_* is the signal strength value that the moving object (PDA) receives from the RF (radio frequency) source *i*. With prior knowledge of position, the distances (length vector) from all APs to mobile nodes are computed. This vector’s component *i* is the real distance from the RF source *i* to the moving object:

(21) $$D={\left[d_{1}d_{2}\ldots d_{5}\right]}^{T}$$

With the help of the Neural Network, [23] we determine the linear regression for vector RSS and the length vector. During online tracking from the received RSS of APs, we used the linear relation previously found by the Neural Network to calculate the distances from the mobile node to APs. We used our Push-Pull estimation [22] to minimize the virtual force-vectors modeled from differences in the currently estimated distance and the measurements.

Even when the learning process is modified with many sets of parameters to make sure the data are not over-fitted, the unstable characteristic is so significant that online tracking does not give good results. The average error (root mean square) of the online tracking is 3.12 m. Figure 5 is the illustration of distance error where the red dot represents a mobile node. This leads to an error in the mobile node’s position estimation.

### 3.4. Context-Aware Activity Manipulation Engine

Ontology is formally defined as an explicit and formal specification of a shared conceptualization [24,25]. Ontology defines formal semantics to allow information to be processable by computer system agents. It defines real-world semantics for resources, allowing them to link machine processable content in a meaningful way based on consensual terminology.

Use of ontology in activity recognition is a new area of research and helps us to better understand an activity in a given context. Researchers have different approaches to context in the recognition process. In [4] the authors only focused on the location and time information for an activity (where context means a lot more than time and location) and use the method of Event-Condition-Action (ECA) to respond to a particular activity. In our approach, we use information about not only the location and time but also the subject profile and the environment in which the subject is currently located.

Activities recognized with the help of different sensors (*i.e.*, body, location, motion, and video sensors) are low level activities and they cannot be used for certain types of analysis and decision making. We use logic to link context information and related activities in a chain. Based on customized rules, we generate higher level results that are more usable for decision making. For instance, low level activities in a series, e.g., bending, sitting, jumping, and walking, can be categorized as exercising. The HARE Repository stores raw data collected from the sensors, the activities recognized by AR engines in XML representation, and the OWL representation of these activities. From the Activity Repository, CAME, with the help of Rules and Inference Engine, manipulates these activities to infer high level activities. To implement CAME with all its components, we have used Jena2, Protégé, Protégé-OWL, Arq, and Pellet 3.4 (for inference). The outcome of CAME is partially dependent on the results of activity recognition modules discussed later. Below is the OWL representation (using N3 notation) of “Walking” in the Activity Repository.

**Table:** 

activityOnto:Activity_Instance_20090614140013345	
a	activityOnto:Activity ;
activityOnto:hasConsequentAction	activityOnto:Action_Instance_145413546;
activityOnto:hasID	345;
activityOnto:hasName	“Walking”;
activityOnto:hasType	“Motion”;
activityOnto:isA	activityOnto:Room_Instance_Class;
activityOnto:performedAtTime	2009:06:14:14:00:13;
activityOnto:performedBy	activityOnto:Person_Instance_345.

**Figure 6.**
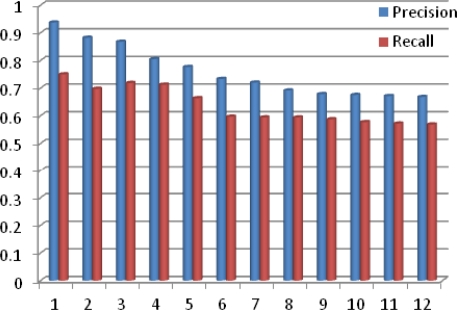
Precision and Recall of CAME for match making *vs.* number of performed experiments.

We have tested CAME using 12 different experiments with increasing numbers of activities, where these activities are all real-time activities detected by different sensors discussed above. We deployed all these sensors in the Test Bed environment shown in Discussion section later. In Figure 6, the y-axis is the % of Precision and Recall for the match making process while the x-axis represents the number of experiments performed. From the graph shown in Figure 6, it is quite obvious that the precision and recall of CAME for the match making process decreases as the number of activities increases, but interestingly, with more experiments, both precision and recall are smoothening. The average precision and recall for CAME match making are 0.759 and 0.636, respectively.

In CAME development, we used an A-Box that only involved instances. We extended CAME and used the integration of A-Box with T-Box. Before applying A-Box, we used T-Box to limit the number of instances by using the structure of the knowledge base. Another main cause for low precision of CAME is the unknown activities detected by the sensors. Since we focus only on a set of 18 activities, any other activity performed by the Alzheimer patient was reported as an “*unknown activity*”. We have also modified CAME for unknown activities by implementing a filter to avoid selecting unknown activities during the match making process, which resulted in better system precision. We still store unknown activities to use for future system enhancement. For instance; when taking a bath there were always two unknown activities one before and one after. After observing the pattern, we determined that locking and unlocking the bathroom door were detected as unknown activities. The precision of extended CAME depends on the sensors deployed to detect human activities in a timely manner.

**Figure 7.**
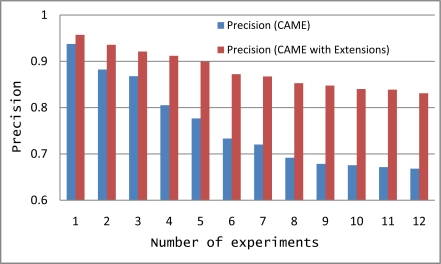
Comparison between CAME and extended CAME precision for 12 different experiments with increasing numbers of activities.

We tested CAME and Extended CAME using the same 12 experiments with increasing numbers of activities. In Figure 7, the y-axis is the % Precision for the match making process while the x-axis represents the number of experiments. The graph in Figure 7 shows that precision of CAME as initially developed is less than the precision of Extended CAME. Though the precision of both are decreasing with an increasing number of activities, extended CAME maintains a good precision rate. Average precision of CAME and extended CAME for 12 experiments are 0.7590 and 0.8810 respectively.

**Rule1**
Exercise ⊑ ∀ Activity ⊓ Activity.performedBy.Person = 1 Person ⊓ (∃Activity.hasContents(bending) ⊔ ∃Activity.hasContents(jacking) ⊔ ∃Activity.hasContents(jumping) ⊔ ∃Activity.hasContents(runing) ⊔ ∃Activity.hasContents(skipping) ⊔ ∃Activity.hasContents(siding) ⊔ ∃Activity.hasContents(walking) ⊔ ∃Activity.hasContents(waving) = ∃ 2 Activity.distinctContents
**Rule2**
∃Activity(a1) ⊓ ┙ hasContents(taking medicine) ⊓ hasNextActivity(a2) ⊓ ∃Activity(a2) ⊓ hasContents(eating) → Activity.Create(a1) ⊓ Activity.Create(a2) ⊓ reminder(take medicine)
**Rule3**
∃Activity(a1) ⊓ hasContents(reading) ⊓ hasNextActivity(a2) ⊓ ∃Activity(a2) ⊓ hasContents(TV On) → Activity.Create(a1) ⊓ Activity.Create(a2) ⊓ turnOff(TV)
**Rule4**
∃Activity(a1) ⊓ hasContents(unknown activity) ⊓ hasNextActivity(null) → Activity.Create(a1) ⊓ reminder(movements are wrong)
**Rule5**
∃Activity(a1) ⊓ hasContents(entering kitchen) ⊔ ∃Activity(a2) ⊓ hasContents(entering bedroom) → Activity.Create(a1) ⊔ Activity.Create(a2) ⊓ turnOn(lights)

The outcome of CAME is highly dependent on the results of the Activity Recognition Engines discussed above. We use two-phase filtering for decision making since using only the results of match making is not sufficient in health care systems. In the second phase we use the description logic rules (see rules above) compiled with the help of knowledge experts (Doctors) to filter the results of the match making process for decision making. Some selected rules used for recommendations and decision making are given. This is not an exhaustive list of all rules. The output of the second phase filter is used for making decisions or suggestions about current situations.

## 4. Discussion

Research on reminder systems for elders to better perform daily life activities [6] is maturing. Most of these approaches are plan-based approaches to decide when and how to prompt subjects effectively, so they focus on time-based activities. To overcome the limitations of this system, a Location-based reminder system has been introduced [7]. However, context for reminders is more important than simple location and/or time. ComMotion [26] is an example of a context-aware system that supports reminder applications. Reminder applications facilitate how and when to prompt subjects. HYCARE [8] is the most recent reminder system that takes context into consideration and uses a novel scheduling mechanism that can coordinate various reminder services and remedy possible conflicts. Compared to the above discussed systems, the proposed system incorporates notions of plan-based and location-based patient activity recognition. Moreover, the proposed system uses patient profile information and medical history for situation analysis and decision making that reduces the risks of missing information.

The system discussed in [4] claims to be a more realistic system. The system uses ontology to incorporate context when processing the collected information. The information is collected from different sensors such as the Smoke Detector, GPRS Modem, Infrared Control and X10 Appliance that facilitate in-home care. It is based on an Event-Condition-Action (ECA) model. However, for support in healthcare, the system also needs to recognize human activities. Our proposed system uses environmental information as well as information on human performed activity to facilitate healthcare while the system discussed in [4] is more a homecare system.

The Mercury [27] system uses different motion sensors to analyze human motion and then based on the recognized activities monitors Parkinson’s and epilepsy patients. Even though, Mercury [27] uses various input modalities for monitoring human activities, the system doesn’t support situational analysis. The Mercury system [27] does not consider context when a patient performs a particular activity. On the other hand, in our proposed system, in addition to different input modalities, the proposed system uses historical information as well as context information to personalize patient response. The positive aspect of Mercury [27] is its low energy consumption and long monitoring duration. However, in a healthcare system, a system must have a mechanism for accurate analysis of a given situation [28]. The system discussed in [29] is the latest Knowledge-Driven approach for human activity recognition based on multiple information streams in a smart home environment. The system uses ontology for context, activity and lifecycle modeling to recognize activities via ADL. The system uses different variations of rules to capture variations in how activities are performed by different users at different times. The system also uses various input modalities of the same type. Motion sensors are vulnerable to faulty inputs or recognizing unintended actions. In our proposed system, different sensors are used to report (in some cases) the same situation that reduces the possibility of faulty input during situation analysis.

Most of the above discussed systems do not use real-time activities or only use one type of real-time activity to generate reminders or make decisions. This results in inflexible system behavior. These systems can mostly be used for reminder systems or home care systems. We seek to perform situational analysis by incorporating the input from different modalities. To facilitate on-demand services and robustness, we have introduced Cloud Computing to reduce cost and improve performance. Existing Cloud-based healthcare systems like those in [30] and [31] do not integrate wireless sensor networks which are necessary to obtain real-time information on the patient and/or environment. Appropriate information dissemination is not explicitly addressed in most research.

Our proposed SC^3^ efficiently uses software and hardware, improves resource utilization and scalability and maintains privacy. The proposed system is a cost efficient model for automating hospitals, managing real-time data from various sensors, efficiently disseminating information to consumers, supporting privacy via a strong authentication mechanism, and appropriately using data to promote health, both within and outside of traditional emergency services.

**Figure 8.**
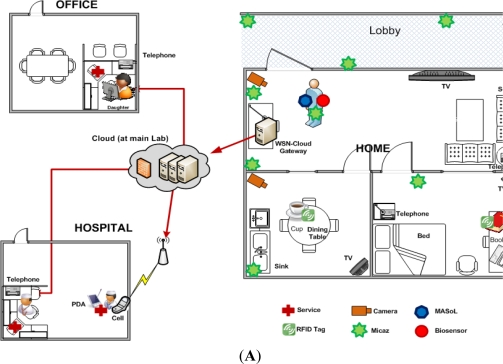

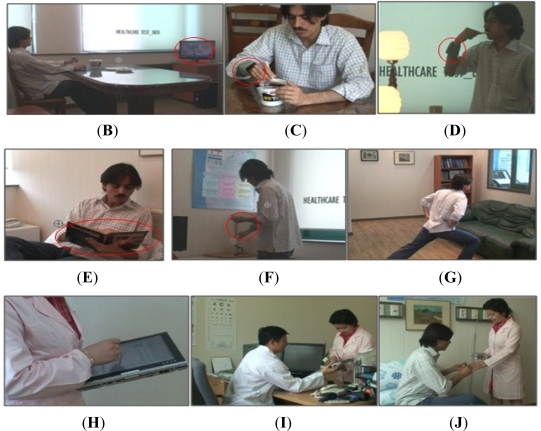
(**A**) Shows the overall scenario designed at a patient home. The other images are the images captured from the video demonstration of SC^3^ for Alzheimer’s patients; (**B**) Shows that patient is sitting and looking towards the TV, SC^3^ detects posture and turns on the TV for him; In **C**, **D**, **E**, and **F**, the body sensor detects that patient is performing eating, tooth brushing, reading, and taking medicine activities. During the reading activity, the system generates a reminder for the patient to exercise and in **G**; the subject is exercising, which is detected by camera-based sensors; In **H**, **I**, and **J**, the doctor and nurse discuss the patient’s condition and revise the patient’s medications.

To deliver the above mentioned services and facilities for Alzheimer patients, we have deployed the SC^3^ system. Our general system deployment design is shown in Figure 8. The patient’s home includes a kitchen, a bed-room, and a living room. We have deployed wearable-sensors, video cameras, and location tracking engines in the patient’s home to collect sensory data and images. We have also deployed motion sensors for simple motion detection. This information is used by a context-aware activity manipulation engine for situation analysis and decision making. We deploy a Cloud gateway in the living room to collect data from all sensors and cameras. It connects to the Cloud via a high speed router. Doctors, nurses, and a patient’s relatives (e.g., his daughter) can access data easily via a Web2.0 interface. In this case study, we haven’t focused on energy use during sensor usage and computation; however, optimized devices and algorithms have been used that capture the data and transmit it to the Cloud server. Computational efficiency has not yet been a focus of our research. One patient participated in the experiments.

We used a WiTilt V3 sensor supported accelerometer and gyroscope attached to the patient’s right hand to detect his activities such as eating, tooth brushing, reading, and taking medicine as shown in Figure 8(B–E), taken from our video demonstration (http://www.youtube.com/watch?v=FfRpsjD3brg) of the system. The details of the scenes given in Figure 8 are described in Table 2. In each room, we deploy a TinyOSMall PIR motion sensor to detect if the patient is in the room. A wide-angle web camera is attached on the wall of the living room and the kitchen to detect his movement such as watching TV or exercising. We installed a free source code Enomaly ECP in 4 PCs Pentium IV dual-core 2.5 GHz, 3 GB RAM to serve as a Cloud server. The scenario for the Alzheimer’s patient is as follows.

At 7 o’clock in the morning, the patient enters the kitchen and has breakfast. When the patient enters, the motion sensor sends a sensed signal to the SC^3^. SC^3^ detects that the patient is in the kitchen, so it sends a command to turn on the light automatically. The patient sits on a chair and is looking at the TV. Information from motion sensors and the location tracking engine gives precise information about the patient’s current location. SC^3^ detects the patient’s posture by collecting image data from the camera and inferring the activity. It sends a command to turn on the TV in the kitchen. Then, SC^3^ collects gyroscope and accelerometer signals from the embodied sensor and infers the patient’s eating and teeth brushing activities (Figure 8(B–D)).

After finishing breakfast, the patient reads a book (detected by embodied sensor) in the bedroom (detected by motion sensor and location sensor). Detecting that the patient is reading, SC^3^ turns off the TV for the patient. A while later, by checking the stored activity knowledgebase, SC^3^ recognizes that the patient did not take medicine and needs to do exercise for today. So it sends a voice reminder “Take medicine please!”, and then “Do exercise please!” to the patient. When the patient performs taking medicine and exercising activities, the actions are detected by embodied sensor and video based AR respectively. Automatically, the knowledgebase is updated to avoid further reminders (Figure 8(E–G)).

At the hospital, the nurse accesses the Cloud and checks the patient’s health condition. She also can see whether the patient forgot to do any routine work such as taking medicine or exercising. If the nurse concludes the patient is not getting better or observes alarms from the system, the nurse discusses the situation with the doctor. After that, the doctor adds a new medication and has the nurse have it delivered to the patient (Figure 8(H–J)).

To implement the system and make appropriate decisions based on the situation, we use the compiled rules given in the Results Section, which are used by the inference engine to make decisions. These rules are not only useful for decision making but are also useful in consistent situation analysis when an activity is performed in multiple ways [29] or by different subjects.

Most of the existing system does have the tendency to behave abnormally when (1) the subject behavior changes or (2) when a different subject uses the system. To overcome the subject behavior issue, we have incorporated the use of rules that help in situation analysis even if the activities are performed in different sequence [29]. For instance: Making Tea activity can be performed in different ways by performing the sub-activities (*i.e.*, sugar use, boiling water, using cup, using tea) in a different sequence. In case when the subject is changed and our system is used for a different subject, it still maintains the same performance. The reason behind it is that we have decomposed the system into generic sub-components and these sub-components overcome all the issues associated with subject change. For instance; the Teeth Brushing activity detection process using the wearable-sensor only considers the actions performed by the subject, not the subject performing the actions. So any subject performing the activity will be recognized by our sub-component. The same way, the other sensors are also not dependent on subject. This generic nature of the sub-component of proposed system helps in maintaining the same performance of overall system for various subjects.

## 5. Conclusions

A framework architecture for a Human Activity Recognition Engine (HARE) has been presented for detecting real-time daily life activities. By using ontologies to model the domain and using expert knowledge, better service provision and more intelligent healthcare facilities have been achieved. Detailed discussion of HARE and its subcomponents is made. The support of HARE for doctors, care-givers, clinics and pharmacies is discussed. In the case study, we observe that the HARE component worked well for an Alzheimer’s patient performing daily life activities.

We are planning to provide more services to patients suffering from kinds of chronic diseases such as stroke, Parkinson’s, and depression. We are also introducing an *i*-LiSP module which uses pattern recognition techniques for smarter service recommendations. This allows the system to learn about the subject and to be more flexible while applying rules.

## Figures and Tables

**Table 1.** Recognition rates with average accuracy = 91.4%. The value of blank cells is 0%.

	**bend**	**jack**	**jump**	**Run**	**side**	**skip**	**walk**	**wave1**	**wave2**
bend	89%								
jack		89%							
jump			78%						
run				100%					
side					89%				
skip			11%			89%			
walk			11%		11%		100%		
wave1								100%	
wave2									100%
Unknown	11%	11%				11%			

**Table 2.** Description of each scene with sensor(s) used and the consequent actions.

**Scene**	**Sensor Used**	**Actions Taken**
A and F	Location and Video Based AR	In scene A, the posture of a subject is detected. The person is sitting and looking towards the TV. Location tracker notes that the person is in the kitchen. The system turns on the TV automatically. In scene F, the video based sensor detects that the subject is exercising and so exercise activity per day is recorded.
B, C, D, and E	Location and Wearable-sensor Based AR	All these activities are detected with the help of a wearable-sensor, but the location tracker gives information about where these activities are performed. All these activities are scheduled activities tracked by the system and afterwards stored in the knowledgebase.
G, H, and I	System Response	In these scenes, the nurse gets an alarm from the system as the patient is having some undefined symptoms (activities). Nurse consults with the doctor and the nurse delivers the new prescribed medicines to the patient’s house.
